# Reduced expression of monocyte CD200R is associated with enhanced proinflammatory cytokine production in sarcoidosis

**DOI:** 10.1038/srep38689

**Published:** 2016-12-08

**Authors:** Simon D. Fraser, Laura R. Sadofsky, Paul M. Kaye, Simon P. Hart

**Affiliations:** 1 Department of Academic Respiratory Medicine, Centre for Cardiovascular and Metabolic Research, Hull York Medical School, Castle Hill Hospital, Cottingham, HU16 5JQ, UK; 2 Centre for Immunology and Infection, Department of Biology and Hull York Medical School, University of York, Wentworth Way, York, YO10 5DD, UK

## Abstract

In sarcoidosis, the proinflammatory cytokines interferon gamma, tumour necrosis factor and interleukin-6 are released by monocyte-derived macrophages and lymphocytes in the lungs and other affected tissues. Regulatory receptors expressed on monocytes and macrophages act to suppress cytokine production, and reduced expression of regulatory receptors may thus promote tissue inflammation. The aim of this study was to characterise the role of regulatory receptors on blood monocytes in patients with sarcoidosis. Cytokine release in response to stimulation of whole blood was measured in healthy controls and Caucasian non-smoking patients with sarcoidosis who were not taking disease modifying therapy. Expression of the regulatory molecules IL-10R, SIRP-α/β, CD47, CD200R, and CD200L was measured by flow cytometry, and functional activity was assessed using blocking antibodies. Stimulated whole blood and monocytes from patients with sarcoidosis produced more TNF and IL-6 compared with healthy controls. 52.9% of sarcoidosis patients had monocytes characterised by low expression of CD200R, compared with 11.7% of controls (p < 0.0001). Patients with low monocyte CD200R expression produced higher levels of proinflammatory cytokines. In functional studies, blocking the CD200 axis increased production of TNF and IL-6. Reduced expression of CD200R on monocytes may be a mechanism contributing to monocyte and macrophage hyper-activation in sarcoidosis.

Sarcoidosis is characterised by increased inflammatory activity within tissue granulomata, with accumulation of activated lymphocytes and monocyte-derived macrophages (epithelioid macrophages) and local release of proinflammatory cytokines^1^,^2^,^3^. Lung macrophages, derived from blood monocytes^4^, are potent producers of TNF and IL-6^5^,^6^,^7^ which contribute to the formation of sarcoid granulomata^8^.

Regulation of inflammatory responses is vital to initiate resolution and prevent excessive tissue damage^9^. Abnormalities of regulatory pathways that normally act to dampen inflammation could explain the hyper-active immunological state seen in sarcoidosis. Interleukin-10 (IL-10) is the archetypal regulatory cytokine involved in control of Th1 immune activity. IL-10 is produced primarily by monocytes and regulatory T lymphocytes, and acts through its receptor IL-10R on T cells, monocytes, and macrophages^10^. Cranshaw *et al*. reported that sarcoidosis blood monocytes produced smaller amounts of IL-10 than controls and were less able to suppress T cell proliferation^11^. Monocytes and macrophages also express signal-regulatory protein-alpha (SIRP-α) which binds to ubiquitously expressed CD47 and acts as an anti-phagocytic signal^12^, and CD200R^13^, which binds its cognate ligand CD200L on many cell types and leads to reduced mitogen-activated protein kinase signalling through recruitment of Ras GTPase activating protein^14^. The CD200R/CD200L axis is vital in maintaining immune homeostasis in the lungs of mice^14^,^15^, and reduced CD200R signalling has been implicated in the pathology of joint inflammation^16^, neurodegeneration^17^, and cancer^18^.

The aim of the present study was to assess the role of regulatory receptors in modulating monocyte cytokine production in sarcoidosis. A whole blood assay was chosen for monocyte stimulation to minimise *ex vivo* perturbation of monocytes. Monocytes are shown to be a source of TNF and IL-6 within stimulated whole blood assays, and monocytes from sarcoidosis patients produced these cytokines to a greater extent than healthy volunteers. Patients with sarcoidosis more commonly had monocytes expressing low levels of CD200R, whereas other regulatory receptors (IL-10R, SIRP-α) were expressed at normal levels. Finally, blockade of CD200R or CD200L led to increased production of TNF and IL-6. Collectively, the data argue that reduced expression of CD200R is an important mechanism underlying monocyte/macrophage hyper-responsiveness in sarcoidosis.

## Results

### Patients with sarcoidosis display T lymphocytopenia

The demographics and clinical details of study participants are shown in Table 1. All subjects were Caucasians. Patients with sarcoidosis were all non-smokers and were not taking corticosteroids or other disease modifying therapies. Twelve subjects with sarcoidosis (40%) had a Scadding stage 0 or 1 chest X-ray (i.e. without visible lung changes) and 18 (60%) had a stage 2 or 3 chest X-ray. All subjects had CT scan evidence of lung parenchymal abnormalities or mediastinal lymph node enlargement. Immunophenotyping of PBMCs showed that patients with sarcoidosis exhibited a general T lymphocytopenia, in keeping with previous reports^19^ (Supplementary Table S3 and Supplementary Fig. S1).

### Sarcoidosis monocytes produce more TNF and IL-6

**Figure 1:**
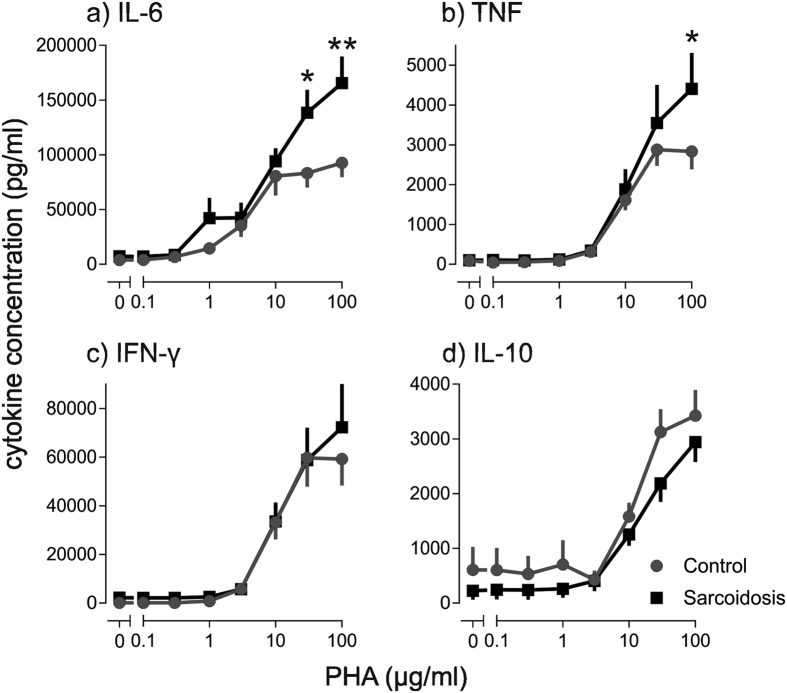
TNF and IL-6 release from PHA-stimulated whole blood is greater in patients with sarcoidosis than healthy controls. Whole blood was stimulated with phytohaemagglutinin (PHA) 0.1–100 μg/ml. (**a**) IL-6 (n = 17), (**b**) TNF (n = 14), (**c**) IFN-γ (n = 21), and (**d**) IL-10 (n = 17) were measured after 16 hours. Results are presented as mean ± SEM; *p < 0.05, **p < 0.001 using two-way ANOVA with Sidak’s *post hoc* test.

**Figure 2:**
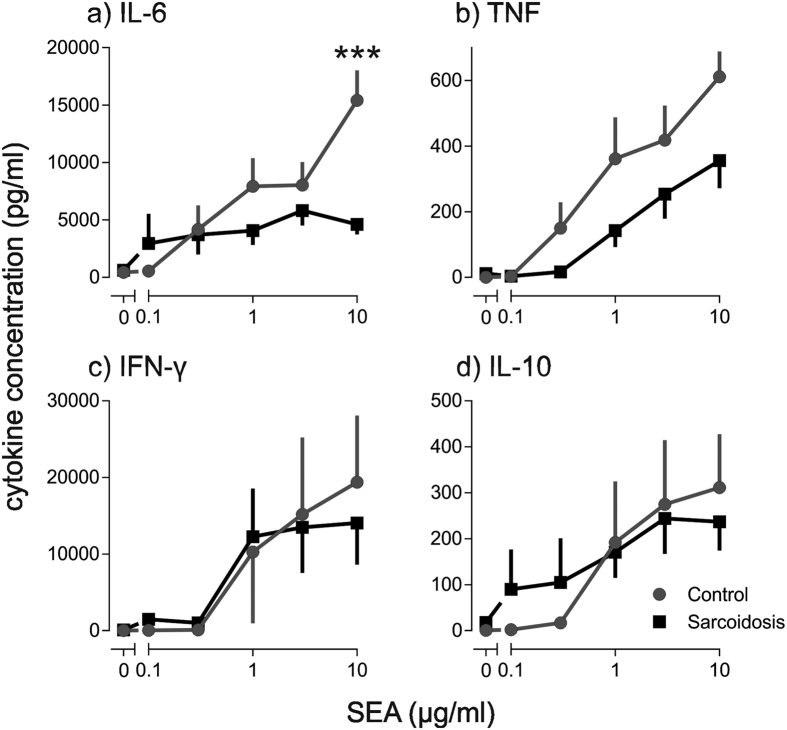
IL-6 release from SEA-stimulated whole blood is reduced in patients with sarcoidosis. Whole blood was stimulated with staphylococcus enterotoxin A (SEA) 0.1–10 μg/ml. (**a**) IL-6 (n = 7–8), (**b**) TNF (n = 6–10), (**c**) IFN-γ (n = 7), and (**d**) IL-10 (n = 5–6) were measured after 16 hours. Results are presented as mean ± SEM; ***P < 0.0001 using two-way ANOVA with Sidak’s *post hoc* test.

A whole blood assay was used for *ex vivo* stimulation studies. Significantly higher concentrations of secreted TNF and IL-6 were found in stimulated whole blood from patients with sarcoidosis compared with healthy controls (Fig. 1). IFNγ and IL-10 were not significantly different between sarcoidosis patients and healthy controls (Fig. 1). When the kinetics of cytokine production in PHA-stimulated whole blood were measured, IFNγ and IL-10 were produced with kinetics commensurate with T lymphocyte activation, whereas TNF production was rapid, peaking at 3–6 hours and declining thereafter (Supplementary Fig. S2). Similar kinetics have been observed by others for monocyte-derived TNF^20^,^21^. PHA is a T cell mitogen^22^ and it also stimulates monocytes by cross-linking Toll-like receptors^23^. To further explore the relative contribution of T lymphocytes and monocytes to the enhanced cytokine release observed in sarcoidosis, whole blood was stimulated with SEA, a more selective T cell mitogen. IL-6 production in response to SEA was significantly lower in blood from sarcoidosis patients (Fig. 2), consistent with the T lymphocytopenia.

**Figure 3:**
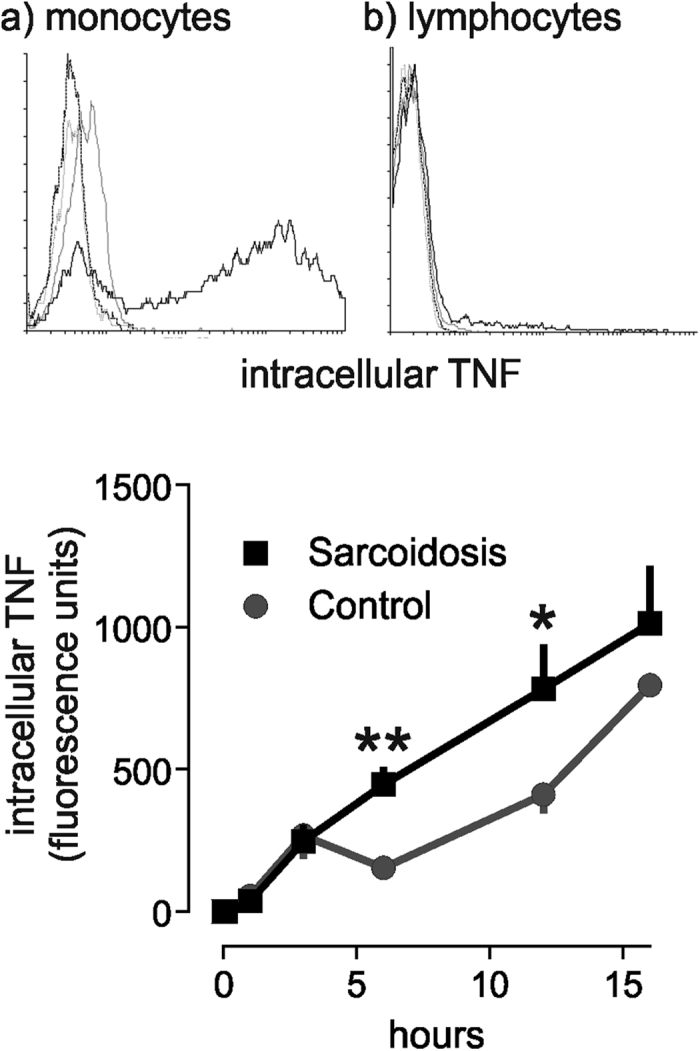
TNF production by monocytes is increased in sarcoidosis. Representative flow cytometry histograms showing intracellular TNF accumulation in (**a**) monocytes and (**b**) lymphocytes after incubation with PHA for 16 hours. Dark grey line, unstimulated isotype control antibody; light grey, unstimulated TNF; solid black, PHA stimulated TNF. x axis, TNF fluorescence intensity; y axis, number of events. (**c**) Intracellular TNF accumulation in monocytes in response to 10 μg/ml PHA in patients with sarcoidosis and controls (n = 3). Results are presented as mean ± SEM geometric mean fluorescence intensity; *p < 0.05, **p < 0.01 using two-way ANOVA with Bonferroni’s *post hoc* test.

To confirm that monocytes were responsible for the enhanced TNF and IL-6 production observed in sarcoidosis patients following PHA stimulation, intracellular accumulation of TNF was quantified by flow cytometry in Brefeldin A-treated PBMCs. PHA led to a substantial accumulation of TNF in monocytes, but not lymphocytes (Fig. 3). In sarcoidosis monocytes, stimulated intracellular TNF accumulation was significantly greater than in healthy controls (Fig. 3). More IL-6 accumulation was also seen in sarcoidosis monocytes (Supplementary Fig. S3).

### Intermediate monocytes are increased in sarcoidosis blood

**Figure 4:**
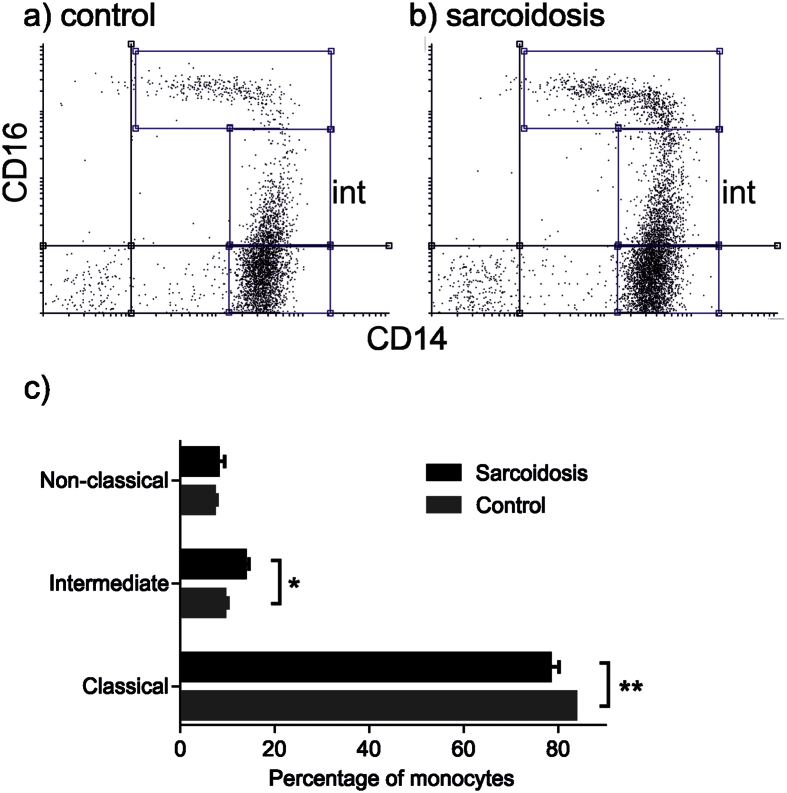
Intermediate CD14^++^ CD16^+^ blood monocytes are expanded in sarcoidosis. Representative two colour flow cytometry dot pots from (**a**) control and (**b**) sarcoidosis subjects with intermediate monocytes indicated (int). Monocyte subsets were determined by extracellular antibody staining for CD14 and CD16. (**c**) Percentages of CD14^++^ CD16^+^ intermediate monocytes, CD14^++^ CD16^−^ classical monocytes, and CD14^+^ CD16^++^ non-classical monocytes in patients with sarcoidosis (n = 14) and controls (n = 18). Results are presented as mean ± SEM; *p < 0.05, **p < 0.01 using two-way ANOVA with Sidak’s *Post hoc* test.

Monocytes appeared to be responsible for elevated TNF and IL-6 responses in sarcoidosis patients, so monocyte subsets were characterised based on relative expression of CD14 and CD16^24^. In sarcoidosis patients there was expansion of the intermediate CD14^++^/CD16^+^ monocyte population (13.6% ± 1.01 (mean ± SEM) compared with 9.3% ± 0.82, p < 0.05) and reduction in classical CD14^++^/CD16^−^ monocytes (78.1% ± 1.13 compared with 83.6% ± 2.01, p < 0.01) (Fig. 4). There was no significant difference in non-classical CD14^+^/CD16^++^ monocytes between the cohorts (7.9% ± 1.51 compared with 7.1% ± 0.76).

### Sarcoidosis patients have reduced CD200R expression on monocytes

**Figure 5:**
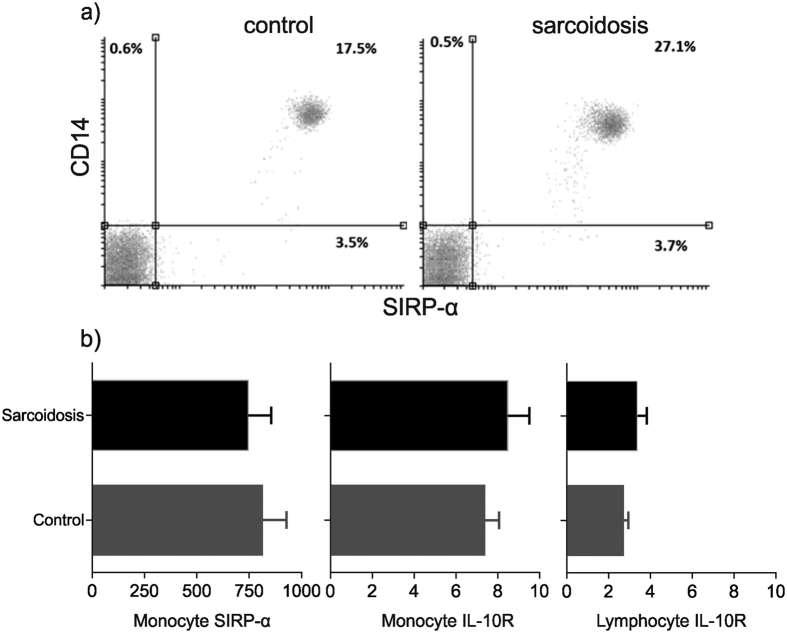
SIRP-α and IL-10R expression is similar in sarcoidosis patients and controls. (**a**) Representative flow cytometry dot plots showing SIRP-α/β expression on monocytes in a healthy control subject and a patient with sarcoidosis. (**b**) Expression of SIRP-α/β and IL-10R on monocytes and T lymphocytes in sarcoidosis patients (n = 9) compared with controls (n = 10). Results are presented as mean ± SEM geometric mean fluorescence intensity; P = NS using Mann-Whitney U test.

Monocyte- and macrophage-derived TNF can be regulated by a variety of cytokines and cell surface receptor-ligand interactions^15^. Therefore, IL-10R, SIRP-α, and CD200R on monocytes were examined to determine whether expression of these receptors was altered in patients with sarcoidosis. Expression profiles of IL-10R and SIRP-α/β were similar in sarcoidosis patients and healthy controls (Fig. 5). Similarly, expression of CD47, the ligand for SIRP-α/β, was the same in sarcoidosis and controls (data not shown).

**Figure 6:**
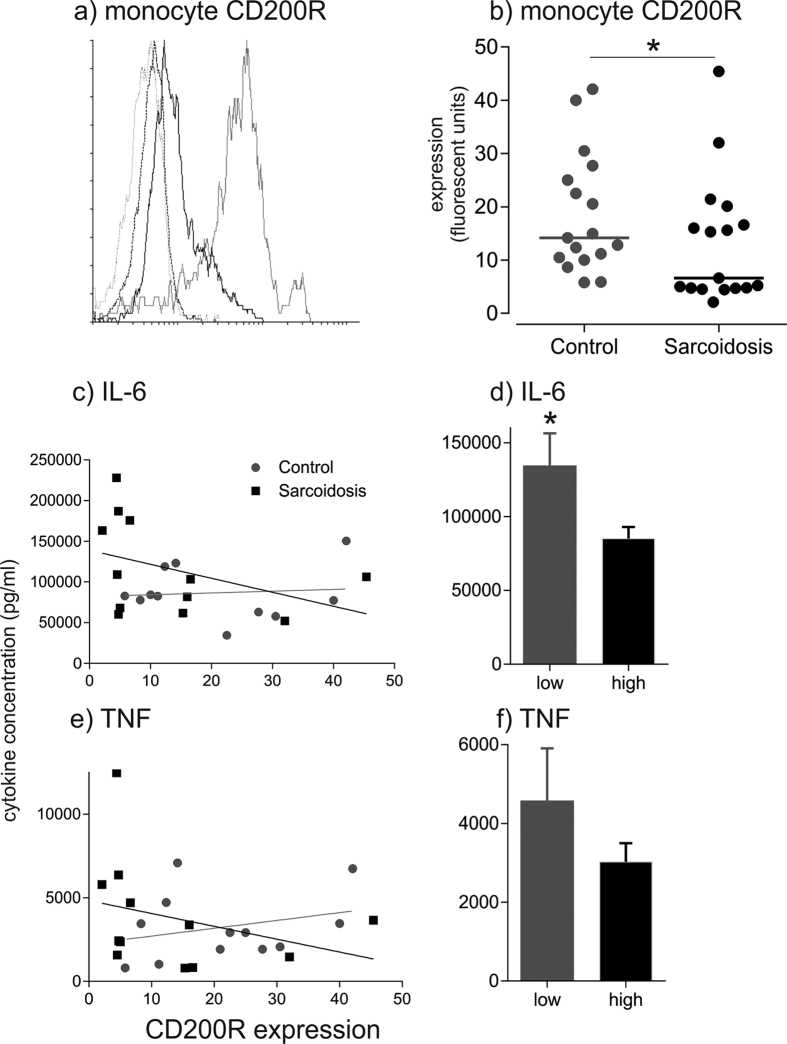
CD200R expression on monocytes is reduced in sarcoidosis, and CD200R^low^ phenotype is associated with increased proinflammatory cytokine release. (**a**) Representative flow cytometry histograms showing CD200R expression on monocytes from a patient with sarcoidosis and a healthy control subject. Grey dotted line, isotype control antibody in healthy control; black dashed line, isotype control in sarcoidosis; grey solid line, CD200R healthy; black solid line, CD200R sarcoidosis. x axis, CD200R fluorescence intensity; y axis, number of events. (**b**) CD200R expression on monocytes in sarcoidosis and control subjects. Medians are indicated by horizontal lines; *p < 0.05 using the Kolmogorov–Smirnov test; n = 17 healthy controls, n = 17 sarcoidosis patients. (**c**) Relationship between monocyte CD200R expression and whole blood IL-6 release in response to stimulation with 100 μg PHA. Black line, sarcoidosis linear regression (n = 13); grey line, control linear regression (n = 10). (**d**) Stimulated IL-6 release in CD200R^low^ (n = 8) and CD200R^high^ (n = 15) pooled subjects; *p = 0.018 using Student’s t-test. (**e**) Relationship between monocyte CD200R expression and whole blood TNF release in response to stimulation with 100 μg PHA. Black line, sarcoidosis linear regression; grey line, control linear regression. (**f**) Stimulated TNF release in CD200R^low^ and CD200R^high^ mixed subjects; p = 0.19.

In contrast, CD200R expression on blood monocytes from patients with sarcoidosis was reduced, with patients clustering as high or low expressers (p < 0.05) (Fig. 6). Using a boundary defining low expression below the standard deviation of the healthy population (geometric mean < 7.28 fluorescence units), 52.9% of sarcoidosis patients were classified as CD200R^low^ compared with 11.7% of healthy donors (P < 0.0001) (Fig. 6). Monocyte CD200R expression was not related to serum ACE activity, C-reactive protein, or plasma viscosity (data not shown). CD200R expression on T lymphocytes in sarcoidosis also polarised as either CD200R^low^ or CD200R^high^ in a bimodal distribution, compared with a unimodal distribution in healthy controls (Fig. S4).

Expression of the ligand CD200L was absent on monocytes, and the percentage of CD200L^+^ T lymphocytes was similar in sarcoidosis patients and healthy subjects (data not shown). However, because of the T cell lymphocytopenia there were fewer T cells expressing CD200L in sarcoidosis blood (0.16 ± 0.03 × 10^6^ compared with 0.39 ± 0.03 × 10^6^ cells/ml, p < 0.0001).

### CD200L/CD200R interactions regulate TNF and IL-6 production

To investigate how CD200L/CD200R interactions regulate proinflammatory cytokine release, monocyte CD200R expression was correlated with stimulated cytokine release in whole blood assays and PBMC cultures. In sarcoidosis, IL-6 responses were significantly higher in subjects with low monocyte CD200R expression when compared with high CD200R expressers (Fig. 6), and a similar trend was observed for TNF. When data from sarcoidosis and healthy subjects were pooled there was a trend towards an inverse correlation between IL-6 release and monocyte CD200R expression (r = 0.39, p = 0.21) (Fig. 6).

**Figure 7:**
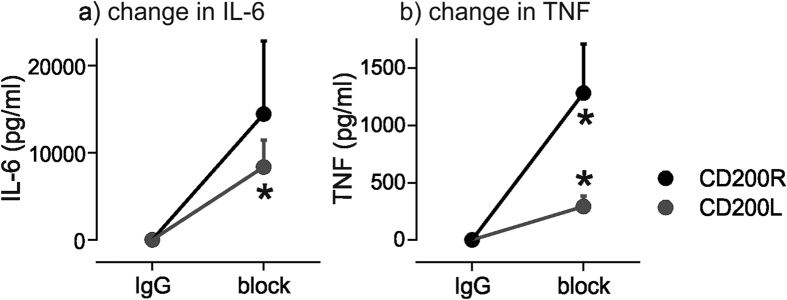
Antibody blocking of CD200R or CD200L in healthy controls increases IL-6 and TNF release. (**a**) Change in IL-6 (pg/ml) in PBMCs and whole blood after incubation with CD200R (n = 5) or CD200L (n = 6) antibodies compared with isotype control IgG. Cells were stimulated with 100 μg/ml PHA for 16 hours. (**b**) Change in TNF (pg/ml) in PBMCs and whole blood after incubation with CD200R or CD200L antibodies compared with isotype control IgG. Cells were stimulated with 100 μg/ml PHA for 16 hours. Results are presented as differences from isotype control IgG; *p < 0.05 using paired t-tests.

To determine whether the CD200L/CD200R axis was important for regulating cytokine production in whole blood, blocking studies were performed using antibodies. CD200R blockade increased cytokine release from isolated PBMCs from healthy subjects (Fig. 7). TNF increased by 35.7 ± 29.2% (mean ± SEM; mean difference 1282 ± 427 pg/ml, P = 0.04), and IL-6 increased by 22.3 ± 19.4% (mean difference 14430 ± 8369 pg/ml, P = 0.16). Similarly, CD200L blockade in whole blood increased TNF and IL-6 release. TNF increased by 14.5 ± 7.4% (mean difference of 292 ± 93.6 pg/ml, P = 0.026) and IL-6 increased by 6.9 ± 3.1% (mean difference 8366 ± 3078 pg/ml, P = 0.042) (Fig. 7).

## Discussion

This study is the first to characterise the expression of the regulatory receptor CD200R and its ligand CD200L on blood mononuclear cells in sarcoidosis. We have demonstrated that the distribution of monocyte CD200R expression in a cohort of patients with sarcoidosis is distinctly different from healthy control subjects, with a significantly higher proportion of sarcoidosis patients exhibiting a CD200R^low^ monocyte phenotype. Low monocyte CD200R expression was associated with heightened TNF and IL-6 production in response to PHA stimulation, and blockade of CD200R or CD200L in healthy controls led to increased TNF and IL-6 secretion by monocytes, recapitulating the hyper-activated monocyte state seen in sarcoidosis.

Monocyte CD200R expression was not associated with serum ACE activity, suggesting that CD200R is not simply a marker of total granuloma burden^25^. Other studies have reported reduced CD200R expression on monocytes in inflammatory arthritis^16^. However, CD200R expression in sarcoidosis did not correlate with serum C-reactive protein or plasma viscosity, signifying that a CD200R^low^ monocyte phenotype is not a manifestation of systemic inflammation. Thus monocyte CD200R appears to reflect a unique aspect of disease activity in sarcoidosis. Further studies are required to assess its value in predicting disease progression and response to anti-inflammatory therapy. Whether a similar hyper-responsive phenotype is exhibited by local tissue macrophages in sarcoidosis has yet to be determined; responses in peripheral blood monocytes may not translate directly to the macrophages within granulomata. Murine models suggest that in lung inflammation, monocytes actively contribute to resident macrophage populations^4^. Lung macrophages are potent producers of TNF and IL-6^5^,^6^ which contribute to the formation of sarcoid granulomata^8^.

Despite the evidence for active cellular immunity within tissue granulomata, previous research on peripheral blood in sarcoidosis has been subject to contradictory reports suggesting immune responses may be enhanced^26^,^27^, diminished^28^,^29^ or similar to healthy populations^30^. Reports of suppressed peripheral blood T lymphocyte responses and skin test anergy to specific antigens and non-specific mitogens may be explained by reduced CD4+ T helper cells or increased regulatory T cells. In the present study, patients with sarcoidosis displayed a reduced peripheral blood immune response when T lymphocytes are selectively stimulated with SEA, consistent with the observed T lymphocytopenia. The increased responsiveness to PHA in sarcoidosis patients was observed particularly at higher concentrations of stimulant, which would be consistent with a dose-dependent model where regulation over a specific threshold is reduced.

The present study implicates blood monocytes as potentially important regulatory cells in sarcoidosis. Cranshaw *et al*. reported that sarcoidosis monocytes had reduced IL-10 production^11^. In the whole blood assay used in the present study, we did not demonstrate that the IL-10 regulatory axis was dysfunctional in sarcoidosis, and the expression profiles of regulatory SIRP-α/CD47 were also similar between sarcoidosis and healthy subjects. The choice of methods for cell isolation and stimulation could explain the differences in the findings, since Cranshaw *et al*. studied monocytes that had been isolated by magnetic bead negative selection and stimulated with lipopolysaccharide^11^. Depending on the nature of the stimulant or other factors, deficiencies in more than one monocyte regulatory pathway may contribute to immune hyper-activation in sarcoidosis. We also demonstrated that in patients with sarcoidosis the intermediate CD16^+^ CD14^++^ blood monocyte population was expanded. Since this monocyte subset has been shown to express the highest levels of the TNF receptor TNFR1^31^, monocytes in sarcoidosis may be more responsive to TNF.

Although the whole blood assay used in the present study has an advantage in minimally perturbing cells such as monocytes that are highly pleiotropic and respond rapidly to changing environments on isolation and in tissue culture, the assay does have some limitations. For example, due to T cell lymphocytopenia, fewer CD200L^+^ lymphocytes will be present in sarcoidosis blood for potential ligation of monocyte CD200R.

Anti-inflammatory drug treatment in sarcoidosis is generally considered to be clinically indicated if, following an initial observation period, there is evidence of progressive disease. Corticosteroid treatment reduces macrophage TNF release and leads to improvement in disease markers, but the effect of steroids on long term outcomes is uncertain and the benefits have to be balanced against the potential for serious side effects^32^,^33^. TNF-blocking biological therapies have been tested, with mixed results. Treatment with the TNF monoclonal antibody infliximab reduced tissue inflammation as measured by ^18^F-fluorodeoxyglucose uptake on positron emission tomography imaging in an open label study^34^, and led to a small (2.5%) improvement in FVC at 24 weeks compared with placebo in patients with chronic pulmonary sarcoidosis in a randomised controlled trial^35^. Conversely, studies with TNF-blocking protein etanercept showed high treatment failure rates^36^, and anti-TNF therapy for rheumatic diseases has been linked in many reports to *de novo* development of sarcoidosis-like granulomata^37^. These findings indicate that therapeutic TNF blockade in sarcoidosis is not a panacea. Alternatively, CD200R is potentially targetable as an anti-inflammatory therapeutic strategy. CD200 recombinant proteins and agonistic antibodies have been reported to reduce macrophage activation and promote immunological tolerance in human cells *in vitro* and in mice *in vivo*^38^,^39^,^40^,^41^.

The present study provides evidence that the CD200R/CD200L axis is important in modulating proinflammatory cytokine release from monocytes in sarcoidosis, and raises the possibility that disease progression could be propagated by reduced monocyte CD200R expression. There is a need for improved treatment options for patients with sarcoidosis, and the CD200L/CD200R axis is potentially tractable for future therapy^42^.

## Methods

### Subjects

Ethical approval for the use of blood samples from healthy volunteers was obtained from the Ethics Committee, Hull York Medical School. Regional ethical approval was obtained to obtain blood and tissue samples from sarcoidosis patients from the Hull and East Riding local research ethics committee (LREC 08/H1304/54). Written informed consent was obtained from all subjects, and all methods were performed in accordance with the relevant guidelines and regulations of Good Clinical Practice. Patients with sarcoidosis were diagnosed by multi-disciplinary assessment and displayed non-caseating granulomata on histopathological analysis. Patients with other granulomatous diseases were excluded. Exclusion criteria for sarcoidosis patients and healthy volunteers were concomitant inflammatory or immunological diseases, treatment with corticosteroids or other immune-modulatory therapies, and current tobacco smoking. Blood was collected in sodium heparin tubes (BD) and processed within 1 hour. The results of each set of experiments informed subsequent experimental protocols so not all patients and controls contributed to all of the experiments. To avoid bias, subjects included in each set of experiments were not selected in any way. The numbers of subjects contributing to each experiment are shown in the figure legends.

### Reagents and Antibodies

See Supplementary Tables S1 and S2.

### Peripheral blood mononuclear cell (PBMC) isolation

Heparinised whole blood was diluted 1:1 with sterile phosphate-buffered saline (PBS) and layered over Histopaque 1077 in a 1:1 ratio. The gradient was centrifuged at 600 × g for 30 minutes without braking. The PBMC layer was removed with a sterile pipette and washed twice before suspending in RPMI medium. Whole blood or isolated PBMCs were incubated at 37 °C in a 5% CO_2_ atmosphere.

### Whole blood cytokine release assay

Phytohaemagglutinin (PHA) or *Staphylococcus aureus* enterotoxin A (SEA) were added to 1 ml aliquots of fresh whole blood in microfuge tubes and incubated at 37 °C for 16 hrs (1–72 hrs for time course experiments). Following centrifugation at 3000 × g for 8 minutes, plasma was collected and stored at −20 °C. Isolated PBMCs were resuspended in the same volume of RPMI media as the original blood volume and incubated under the same conditions as whole blood. ELISA kits (Biolegend, San Diego, USA) were used to measure IFN-γ, IL-10, TNF, IL-6, IL-4 and IL-12(p70) according to the manufacturer’s instructions. Plates were analysed using a Multiskan FC Microplate Photometer (Thermo Scientific, Waltham, USA). Results below the minimum detectable limits with 1:2 dilutions of plasma were recorded as zero.

### Immunophenotyping by flow cytometry

PBMCs (2 × 10^5^ cells) were suspended in 50 μl of FACS blocking solution containing antibody (10 μg/ml) and incubated at 4 °C in the dark for 1 hour. Cells were washed and resuspended and analysed using a FACSCalibur flow cytometer and Cellquest Pro software (BD, Oxford, UK). Additional analyses were performed using Flowing Software 2.5.0 (Perttu Terho). Gates were defined using isotype control antibodies.

### CD200R/CD200L blocking assay

Whole blood or PBMCs from healthy donors were incubated with antibodies against CD200L, CD200R, or isotype control (10 μg/ml) or PBS for 1 hour at 37 °C, prior to addition of 100 μg/ml PHA. Supernatants were collected after 16 hours and stored at −20 °C prior to ELISA.

### Cytokine measurement by intracellular flow cytometry

PBMCs (2 × 10^5^ cells) in 96 well round bottom plates were treated with Brefeldin A to ensure cytokine accumulation for 1–16 hrs. Cells were blocked for 1 hour, incubated with fluorophore-conjugated CD3 or CD14 antibodies for 1 hour on ice, fixed with 4% paraformaldehyde, permeabilised with ice cold 90% methanol for 30 minutes, and blocked. Intracellular staining with directly conjugated IL-6, IL-10, TNF, IFNγ antibodies or isotype control IgG (10 μg/ml) was performed at 4 °C for 1 hour followed by flow cytometric analysis.

### Statistical analysis

Sample data were analysed for normality using a D’Agostino and Pearson omnibus normality test or Shapiro-Wilk normality test as appropriate. Statistical analyses were performed using GraphPad Prism (GraphPad Software, La Jolla, USA). Statistical significance was determined using Mann-Whitney U, Kolmogorov–Smirnov, or Kruskal-Wallis tests for non-parametric distributions and Student’s T-test, one-way ANOVA or two-way ANOVA for parametric distributions. Multiple comparisons underwent Sidak, Tukey’s or Bonferroni post-hoc analyses as appropriate.

## Additional Information

**How to cite this article**: Fraser, S. D. *et al*. Reduced expression of monocyte CD200R is associated with enhanced proinflammatory cytokine production in sarcoidosis. *Sci. Rep.* **6**, 38689; doi: 10.1038/srep38689 (2016).

**Publisher's note:** Springer Nature remains neutral with regard to jurisdictional claims in published maps and institutional affiliations.

## Supplementary Material

Supplementary Information

## Figures and Tables

**Table 1:** Demographics and clinical data for healthy subjects and patients with sarcoidosis.

Category	Healthy	Sarcoidosis
Number	31	30
Age (years)	41 (20–72)	51 (28–73)
Male/female	13/18	16/14
Scadding chest x-ray stage (%)	—	
0		7
1		33
2		43
3		17
Serum ACE activity (reference range 8–65 U/L)	—	84.5 (25–356)
C-reactive protein (reference range 0–8 mg/L)	—	3.8 (0.5–64)
Plasma viscosity (reference range 1.5–1.72 m.Pas.s)	—	1.68 (1.49–1.93)

Data are presented as median (range).
