# Supplementary material for: Reduced expression of monocyte CD200R is associated with enhanced proinflammatory cytokine production in sarcoidosis
Source: Sci Rep. 2016 Dec 8;6:38689. doi: 10.1038/srep38689 (PMC5144133; doi:10.1038/srep38689)
Supplement: Supplementary Information [file srep38689-s1.pdf]

Supplementary information

Reduced expression of monocyte CD200R is associated with enhanced proinflammatory cytokine production in sarcoidosis

Simon D. Fraser [1], Laura R. Sadofsky [1], Paul M. Kaye [2], Simon P. Hart [1*]

[1] Department of Academic Respiratory Medicine, Centre for Cardiovascular and Metabolic Research, Hull York Medical School, Castle Hill Hospital, Cottingham, UK, HU16 5JQ

[2] Centre for Immunology and Infection, Department of Biology and Hull York Medical School, University of York, Wentworth Way, York, UK, YO10 5DD

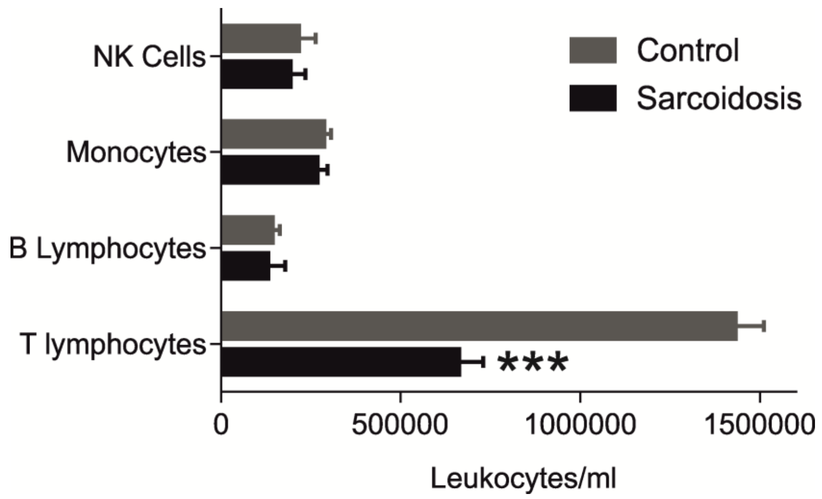

Figure S1. **Peripheral blood mononuclear cell subsets in sarcoidosis subjects demonstrate a T lymphocytopenia.** T lymphocytes ($CD3^+$), B lymphocytes ($CD19^+$), monocytes ($CD14^+$) and Natural Killer cells ($CD3^-CD56^+$ lymphocyte gated) were determined by flow cytometry. Data are presented as mean±SEM; using one-way ANOVA with Tukey's *post hoc* test. B-lymphocytes and NK-cells n=10, healthy control T-lymphocytes and Monocytes n=25, sarcoidosis patients T-lymphocytes and monocytes n=22.

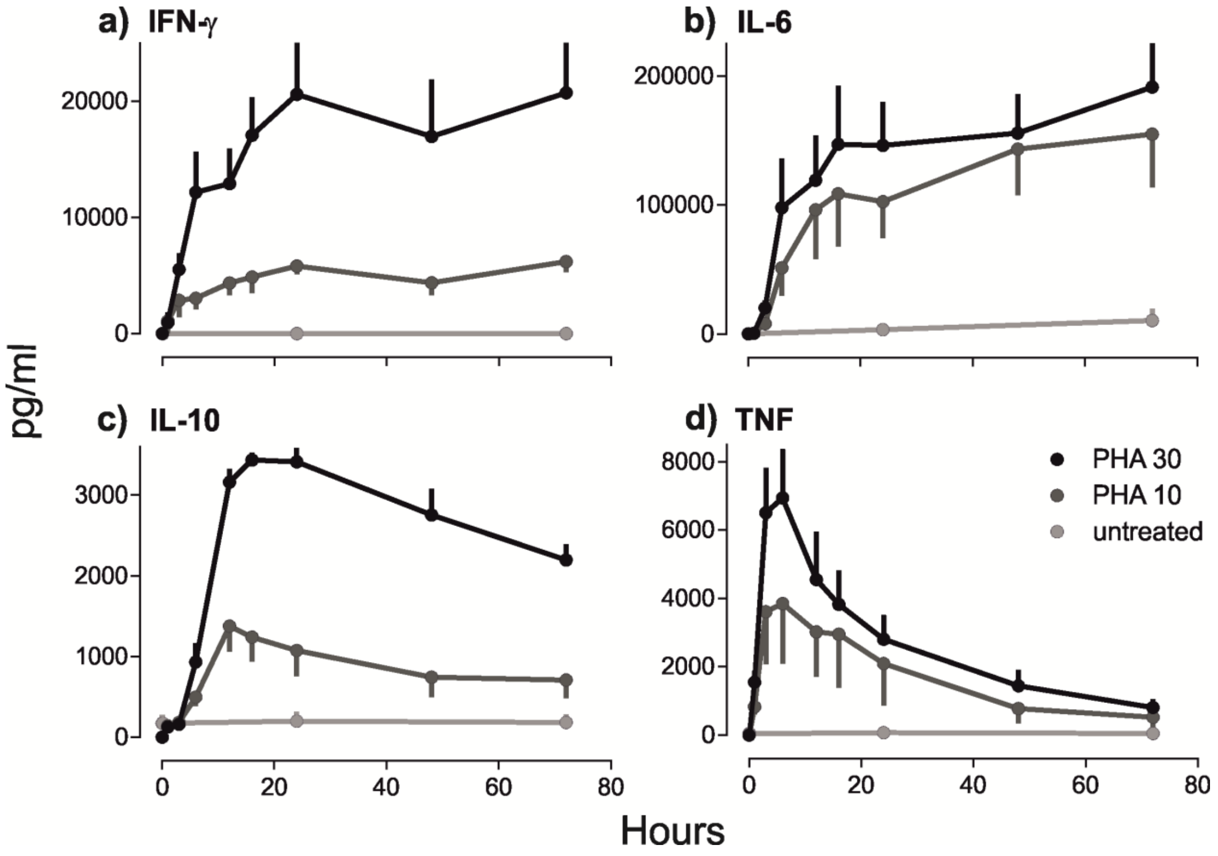

Figure S2. **Cytokine release profiles from whole blood show TNF is produced early.** Whole blood was incubated with PHA (10, 30 µg/ml) or left untreated for up to 72 hours. Plasma was collected at intervals for measurements of a) IFN-γ, b) IL-6, c) IL-10, and d) TNF. Results are presented as mean±SEM; n=4 healthy controls.

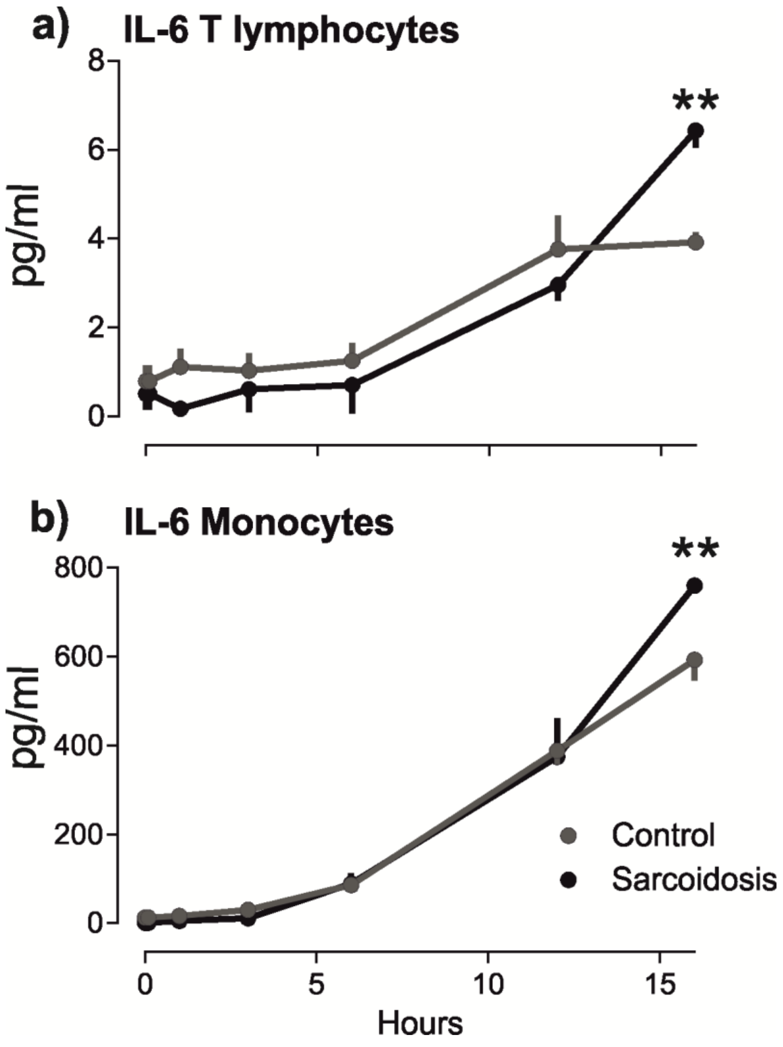

Figure S3. IL-6 is produced primarily by monocytes. a) Intracellular accumulation of IL-6 in T-lymphocytes. b) Intracellular accumulation of IL-6 in monocytes. PBMCs were treated with 5µg/ml of Brefeldin A and 10µg/ml of PHA. Results are presented as mean±SEM; **= p<0.01 using two-way ANOVA. healthy controls n=3, sarcoidosis patients n=2.

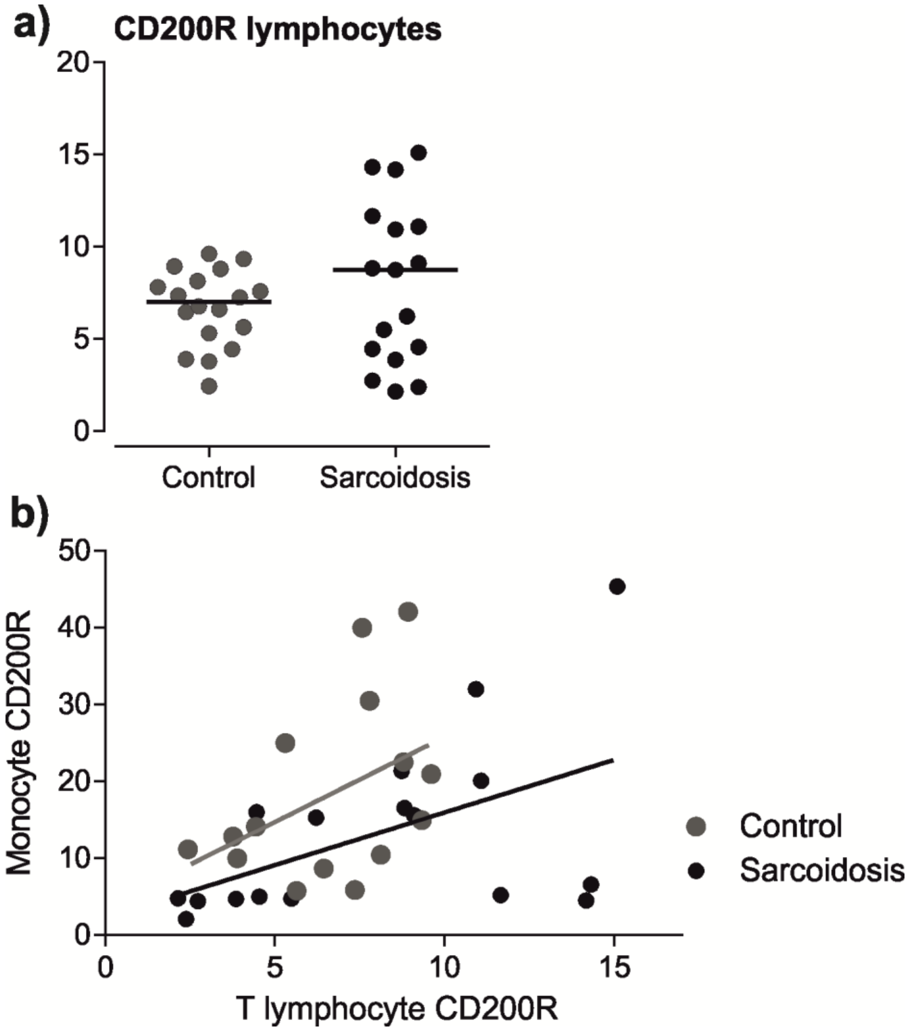

Figure S4. CD200R expression on T lymphocytes is polarised as low or high expressing populations in sarcoidosis. a) CD200R expression on T lymphocytes (geometric mean fluorescent units) in patients with sarcoidosis and healthy controls. P=NS using Kolmogorov–Smirnov test. b) Relationship between CD200R expression on T lymphocytes and monocytes within individual subjects. Grey line = linear regression for healthy controls, R^2=0.19; black line = linear regression for sarcoidosis patients, R^2=0.26. Healthy controls n=18, sarcoidosis patients n=17

Reagents

RPMI 1640 media (Lonza) was supplemented with 10% heat inactivated foetal bovine serum (Gibco) and 100 units/ml penicillin and 100 µg/ml streptomycin (Lonza). Histopaque 1077 (Sigma) was used for separation of PBMCs from whole blood. FACS Blocking Solution was made using PBS and 0.5% protease free high grade bovine serum albumin (Fisher Scientific). ELISA materials not supplied with kits included the ELISA wash buffer (0.05% Tween 20 in 1xPBS (Fisher Scientific)), assay diluent (1% BSA in 1xPBS), 96 well Nunc Maxisorb plates (Fisher Scientific), 1M Sulphuric Acid (VWR) and TMB substrate solution (Vector Laboratories). Phytohaemagglutinin as PHA-P was purchased from Sigma Aldrich and *Staphylococcus* enterotoxin A (SEA) was obtained from Sigma-Aldrich, both were reconstituted and stored as per the manufacturer's instructions. All antibodies for immunophenotyping were obtained from Biolegend and were mouse monoclonal with accompanying matched isotype controls (see Table E1). Antibodies used in intracellular flow cytometry and regulatory receptor characterisation were obtained from Biolegend (see Table E2). CD200 receptor blocking antibody was obtained from R&D Systems, anti-human CD200 R1 polyclonal goat antibody and the control normal goat polyclonal IgG control. CD200 blocking antibody was obtained from R&D systems, human CD200 IgG1 (clone # 325531) and isotype control from Biolegend Human LEAF IgG1.

Target Protein	Host Species	Isotype	Fluorophore	Clone	Reference Number
CD3	Mouse	IgG1, κ	APC	UCHT1	300412
CD4	Mouse	IgG1, κ	APC	RPA-T4	300514
CD8a	Mouse	IgG1, κ	PE	HIT8a	300908
CD14	Mouse	IgG1, κ	APC	HCD14	325608
CD14	Mouse	IgG1, κ	FITC	HCD14	325604
CD14	Mouse	IgG1, κ	PE	HCD14	325606
CD16	Mouse	IgG1, κ	APC	B73.1	360706
CD25	Mouse	IgG1, κ	FITC	BC96	302604
CD45RA	Mouse	IgG2b, κ	FITC	HI100	304148
CD45RO	Mouse	IgG2a, κ	PE	UCHL1	304206
Isotype Control	Mouse	IgG1, κ	APC	MOPC-21	400122
Isotype Control	Mouse	IgG1, κ	FITC	MOPC-21	400110
Isotype Control	Mouse	IgG1, κ	PE	MOPC-21	400114
Isotype Control	Mouse	IgG2b, κ	FITC	MPC-11	400307
Isotype Control	Mouse	IgG2a, κ	PE	MOPC-173	400212

Table S1

Target Protein	Host Species	Isotype	Fluorophore	Clone	Reference Number
IFN-γ	Mouse	IgG1, κ	FITC	4S.B3	502506
IFN-γ	Mouse	IgG1, κ	PE	B27	506507
IL-6	Rat	IgG1, κ	PE	MQ2-13A5	501107
IL-10	Rat	IgG1, κ	PE	JES3-9D7	501404
TNF-α	Mouse	IgG1, κ	PE	Mab11	502909
Isotype Control	Mouse	IgG1, κ	FITC	MOPC-21	400138
Isotype Control	Mouse	IgG1, κ	PE	MOPC-21	400140
Isotype Control	Rat	IgG1, κ	PE	RTK2071	400408
CD47	Mouse	IgG1, κ	FITC	CC2C6	323106
CD172a/b (SIRPα/β)	Mouse	IgG1, κ	PE	SE5A5	323806
CD200 (OX2)	Mouse	IgG1, κ	PE	OX-104	329206
CD200 Receptor	Mouse	IgG1, κ	PE	OX-108	329306
CD210 (IL-10R)	Rat	IgG2a, κ	PE	3F9	308804
Isotype Control	Mouse	IgG1, κ	FITC	MOPC-21	400110
Isotype Control	Mouse	IgG1, κ	PE	MOPC-21	400114
Isotype Control	Rat	IgG2a, κ	PE	RTK2758	400508

Table S2

**Leukocyte Population**	**Healthy**	**Sarcoidosis**	**P value**
PBMC number ($x10^6$ cells/ml)	2.03 (0.60)	1.21 (0.40)	< 0.0001
Percentage of monocytes in PBMCs	15.1 (4.84)	22.2 (8.49)	0.001
Lymphocyte:monocyte ratio	4.67 (2.58)	2.34 (1.50)	< 0.0001
Percentage of $CD3^+$ T lymphocytes in total lymphocytes	72.6 (6.42)	54.4 (15.00)	< 0.0001
Ratio of $CD4^+$:$CD8^+$ T lymphocytes	1.93 (0.99)	2.28 (2.24)	0.561
Ratio of naïve to memory T lymphocytes (CD45RA:RO)	0.86 (0.63)	0.58 (0.50)	0.185
Percentage of $CD19^+$ B lymphocytes in PBMCs	8.53 (4.40)	11.6 (8.80)	0.679
Percentage of $CD3^-CD56^+$ Natural Killer cells in PBMCs	11.9 (5.46)	18.5 (6.75)	0.778
Percentage of $CD3^+CD56^+$ NK-like T lymphocytes in T lymphocytes	7.70 (3.01)	5.24 (3.39)	0.190
Percentage of $CD25^+$ activated T lymphocytes in T lymphocytes	22.2 (6.49)	28.8 (11.0)	0.025

Table S3. Leukocyte populations in subjects with sarcoidosis and healthy controls. Results are presented as mean±SD and analysed by Student’s T-test or Mann-Whitney U test (controls n= min 5, max 21, sarcoidosis patients n= min 5, max 19).
